# Current Developments in the Use of Biomarkers for Juvenile Idiopathic Arthritis

**DOI:** 10.1007/s11926-013-0406-3

**Published:** 2014-01-21

**Authors:** Chantal L. Duurland, Lucy R. Wedderburn

**Affiliations:** 1 Rheumatology Unit, Institute of Child Health, University College London, London, UK; 2 Centre for Adolescent Rheumatology at University College London, University College London Hospital and Great Ormond Street Hospital, London, UK; 3 UCL Institute of Child Health, 30 Guilford Street, London, WC1N 1EH UK

**Keywords:** Biomarker, JIA, Juvenile idiopathic arthritis, CRP, ESR, S100, MRP, SNP, MTX, Methotrexate, Treatment, Remission

## Abstract

Use of biomarkers in clinical practice has proved extremely valuable and is a rapidly expanding field. However, despite the huge potential of biomarkers, for juvenile idiopathic arthritis (JIA) there are currently no validated paediatric biomarkers available to help with setting up a more tailored approach on which drug choice could be based, to achieve remission early in the course of disease. Early remission reduces burden of disease, limits side effects from toxic and unnecessary medication, and, most importantly, enhances quality of life. Several studies have suggested promising biomarkers: these may be a protein, cellular component, mRNA, or genetic component, for example a single nucleotide polymorphism (SNP). Here we describe recent developments in the use of biomarkers for JIA and their potential to assist in management of disease by predicting disease phenotype, severity, progression, and response to treatment, and determining when patients have reached stable remission and can safely discontinue treatment.

## Introduction

Juvenile idiopathic arthritis (JIA) is the most common inflammatory rheumatic disease in childhood, affecting one in 1000 children [1]. JIA is characterized by severe joint inflammation in one or more joints, which persists for at least six weeks, with disease onset before the age of 16. This heterogeneous group of diseases can be divided into several subtypes on the basis of clinical symptoms, medical history, and abnormalities in laboratory measures [2].

A biomarker is a small component which is easily measurable in accessible patient material, e.g. blood, urine or saliva, and is ideally obtained using a relatively non-invasive approach. Ideally, the component used as a biomarker should be stable over time within the sample, and would be able to be measured by an accurate, reproducible assay, at a relatively affordable cost to health service providers. In addition, the ideal biomarker for paediatric use would not be affected by age-related development of children, avoiding the need for age-specific normal-range data sets. Biomarkers are already used in many areas of clinical practice, but most biomarker studies focus on adults rather than children. Data from these studies are sometimes extrapolated to children without considering differences in disease pathogenesis, age-dependent changes in reference ranges for biological laboratory measures, growth and development of children over time, effect of ontogeny on disease evolution and response to treatment, and changes in phenotypic gene expression [3, 4]. Despite the huge potential of paediatric biomarkers, for JIA there are currently no validated paediatric biomarkers available to help in setting up a tailored or “personalised” approach on which drug choice can be based. A more tailored approach would be beneficial for patients because it could facilitate disease remission at an earlier disease stage, which would reduce burden of disease, limit side effects, and improve quality of life [5].

In this review we will discuss recent developments in the potential use of biomarkers for JIA, for predicting the type, severity, and progression of disease after onset and the development of complications linked to JIA, and their effect on our ability to predict response to treatment and determine when stable disease remission has been reached.

## Use of Biomarkers to Predict Disease Phenotype, Severity, and Progression

JIA is a heterogeneous group of disorders, and its classification relies on both clinical findings and a small number of biomarkers used to divide cases into relatively homogeneous subtypes [2]. For example, the two polyarticular forms of JIA, involving five or more joints in the first six months of disease, are distinguished by the absence or presence of serum autoantibody, known as rheumatoid factor (RF), on two occasions three months or more apart. These two clinical subgroups of JIA are distinct in their genetics, age of onset, and prognosis [6, 7]. Similarly, the presence of positive serum anti-nuclear antibody (ANA) has been revealed in several studies to be associated with an increased risk of chronic anterior uveitis in JIA. This is a serious comorbidity of JIA, involving painless but potentially very damaging inflammation of the anterior chamber of the eye, which requires assiduous screening to prevent permanent visual loss [8, 9]. A recent large cohort study has confirmed earlier studies demonstrating that ANA positivity is a risk factor for developing JIA-associated uveitis [10]; current UK clinical guidelines for how long screening should be continued include the use of ANA positivity to direct clinical practice [11]. Thus, both of these antibody biomarkers (RF and ANA), which are stable proteins and are easily measured in a small volume of serum, form part of routine clinical care and treatment pathway decisions for JIA.

At the mild end of the JIA clinical spectrum, oligoarticular JIA, which presents with involvement of four or fewer joints in the first six months of disease, can lead to widely divergent outcomes, ranging from complete remission off medication, to a more severe, extended form of JIA which spreads to involve many joints. Extended oligoarticular JIA can be highly erosive and destructive and may be difficult to control with conventional disease-modifying antirheumatic drugs (DMARDs), frequently requiring long-term treatment with biological therapy [12]. Several studies have revealed that immunological differences between these two outcomes (persistent versus extended oligoarticular JIA) can be observed in the inflamed joint. For example, T cell types (e.g. regulatory T cells, or highly proinflammatory Th17 cells) and their frequencies differ significantly between these two clinical types [13, 14]. In addition, differences in cell frequencies, inflammatory protein levels, and gene expression can be detected in children who will develop more severe disease, before extension occurs: for example, in the so-called “extended-to-be” group of cases, the CD4:CD8 ratio in synovial fluid is lower and the levels of the chemokine CCL5 are higher in extended-to-be oligoarticular JIA compared with persistent oligoarticular JIA [15•]. In this study, analysis of differentially expressed genes at mRNA level also provided novel insights into the pathological mechanisms involved in severity, indicating the importance of the complement pathway and of activated monocytes in extension to more severe disease.

Studies analysing the proteome within synovial fluid have also revealed differences between these subtypes: Gibson et al. demonstrated that the proteome and post-translational modifications of proteins differed between those whose disease remained mild and those who went on to develop severe disease [16]. Validation of these findings will be required before development of a predictive biomarker test, but this field may well yield valuable biomarkers by which to identify children with a poor prognosis.

Assessment of disease activity in JIA typically includes measurement of inflammatory markers in peripheral blood, including either the erythrocyte sedimentation rate (ESR) or C-reactive protein (CRP) [17, 18]. Both of these inflammatory indices have low sensitivity and specificity, because they can be raised for many reasons other than JIA activity and in some children do not closely mirror disease activity. Recently, the pro-inflammatory S100 proteins, S100A8/9 (also known as calprotectin or myeloid-related protein (MRP) 8/14) and S100A12, have been described to be sensitive measures for disease activity in JIA, and both correlate well with physicians’ assessment of disease or with actively inflamed joints [5, 19]. S100 proteins can be measured in serum by enzyme-linked immune-sorbent assay (ELISA). However, the standardisation of the measurements and the detailed procedures for assay and dilution of serum samples from a wide range of patients, with varying disease activity, are non-trivial, complicating implementation of such a test in routine clinical care.

The S100 proteins are released into serum at highly elevated levels in the most severe form of JIA, systemic onset JIA (sJIA), where they correlate well with disease activity—as assessed by physicians’ global assessment of disease activity (*r* = 0.62), Childhood Health Assessment Questionnaire (*r* = 0.56), and active joint count (*r* = 0.46)—and with CRP (*r* = 0.71) and ESR (*r* = 0.72) (for all *p* < 0.001) [20]. A recent study which included measurement of serum S100 proteins has proposed a biomarker panel for predicting flare in sJIA, compared with quiescent disease [21]. The same group also tested the feasibility of measuring such biomarkers in urine for sJIA, which could have many advantages for the paediatric population, especially if testing “kits” could be designed for use in the clinic office or even at home [22••].

One of the most severe complications of sJIA, known as macrophage activation syndrome (MAS) or secondary hemophagocytic lymphohistiocytosis (HLH), remains a cause of mortality in JIA and may be difficult to distinguish from infection [23, 24•]. A gene expression profiling study, of sJIA patients with or without MAS compared with control patients, identified clusters of genes that correlated with sJIA activity and with MAS. If validated, these gene clusters could lead to an mRNA-expression biomarker panel for predicting this potentially life-threatening complication, or to distinguish it from other complications. Interestingly, some of the differentially expressed mRNA species identified, which differed between children with MAS and those without, were transcripts of genes known to be involved in other causes of HLH, for example RAB27a and SH2D1A [25].

Thus, several autoantibodies are already in widespread use as biomarkers in routine care of JIA, and some newer biomarkers (protein, cellular, or mRNA) are under development, for use in defining disease subtype, probable disease course, or comorbidity.

## Use of Biomarkers to Predict Response to Treatment

Recommendations for treatment of JIA are made by the American College of Rheumatology (ACR), and are regularly reviewed. Depending on the number of joints affected—and after failure of monotherapy with non-steroidal anti-inflammatory drugs (NSAIDs) and/or glucocorticoids joint injection in cases of less severe disease—the first-line treatment approach for active disease is administration of methotrexate (MTX). In cases of no or poor response to MTX, biological agents, for example drugs which block tumour necrosis factor alpha (TNFα), are added to the treatment strategy [26]. Although many patients respond well to MTX and reach stable disease remission, approximately 30–50 % [27–29] of patients treated with MTX either do not respond or respond poorly. Many studies analysing response to MTX used the core set variables defined by Giannini et al. [17], in which levels of response in terms of ACR30, 50, or 70 are calculated. Those patients who fail to respond to MTX are first exposed to MTX, and experience side effects associated with non-response to MTX, before more effective biological agents can be offered [5]. A biomarker (or set of biomarkers) predicting which patients will respond to treatment would be beneficial for the patient and prevent side effects caused by ineffective drugs.

The pro-inflammatory S100 proteins have been shown to correlate well with disease activity [5, 19, 20, 30], and recently the level of S100 proteins in serum has been shown to also be correlated with response to treatment. Six months after starting treatment, MRP8/14 levels were lower in sJIA patients responding to MTX treatment (defined as reaching at least ACR70), whereas MRP8/14 levels in sJIA patients not responding to MTX treatment (ACR30) were increased or slightly decreased. Furthermore, sJIA patients treated with anti-IL1 or anti-TNFα had reduced MRP8/14 levels and disease activity [20]. Patients who did respond to MTX had more active joints, higher CRP levels, and higher serum cytokine levels before the start of MTX, which suggests that higher disease activity is positively correlated with good response to MTX. In addition, JIA patients with high levels of MRP8/14 before starting MTX treatment had a higher chance of good response to MTX and had better disease outcomes (at least ACR50) after six months of treatment than non-responders (ACR30 or below) [5].

Differences between patients and response to treatment could be genetic, and therefore genetic factors could be potential biomarkers. Several early studies have suggested associations between genetic polymorphisms and response to MTX. One study identified two single nucleotide polymorphisms (SNPs) in the 5-aminoimidazole-4-carboxamide ribonucleotide transformylase (ATIC) gene and one SNP in the inosine triphosphate pyrophosphatase (ITPA) gene which were found to be associated with a higher risk of poor response to MTX treatment. Using a validation cohort, one of the SNPs in the ATIC gene had a trend towards association with response to MTX [31]. Genotyping of SNPs in genes involved in the polyglutamylation process of MTX and in cellular uptake and efflux of MTX have also shown association with MTX response. One SNP in the adenosine-triphosphate-binding cassette transporter B1 (ABCB1) and one SNP in ABCC3 were found to be associated with good response to MTX, whereas an SNP in the solute carrier 19A1 was associated with poor response to MTX [32].

In another approach, SNP genotyping of genes found to be differentially expressed in a gene expression profiling study, of a UK cohort of JIA patients before and after treatment with MTX, identified three SNPs in the solute carrier family 16 member 7 (SLC16A7) gene associated with response to MTX. Validation of these SNPs in a validation cohort revealed significant association of one of the SNPs with non-response to MTX [33].

A prediction model was recently developed combining clinical and genetic variables to predict non-response of JIA patients to MTX. In this study, response was defined as reaching ACR70 in at least two out of three visits during the first year of treatment. Such a model is important, because it could help in preventing unnecessary treatment of patients who will benefit from monotherapy with MTX and do not need additional treatment with biologicals, which are expensive and might have side effects. The clinical variable included in the model is the ESR. Genetic variables are SNPs in the genes coding for methionine synthase reductase, multidrug resistance 1 (MDR-1/ABCB1), multidrug resistance protein 1 (MRP-1/ABCC1), and proton-coupled folate transporter (PCFT). The model had a moderate predictive power of 65 % for the validation cohort, which might be because of the small number of patients included in the validation cohort [34•].

Thus, evidence suggests that the pro-inflammatory S100 proteins have the potential to serve as biomarkers for predicting response to treatment. This now needs validation in the clinic. Furthermore, several SNPs have been found to be associated with response to treatment, indicating that genetic factors can also provide such biomarkers. It is probable that a combination of genetic, biological, and clinical variables will be required to develop accurate and robust predictive algorithms with which to predict response to drug treatment of JIA, and these may need to be disease-subtype specific.

## Use of Biomarkers to Predict When Stable Remission is Reached

Clinical remission in JIA can be reached with the use of medication [35], and is ideally maintained after treatment is stopped [1] (criteria for clinical remission on and off treatment are described by Wallace et al. [36]). However, after discontinuation of MTX treatment, approximately 30–50 % of patients relapse [27–29], suggesting the presence of subclinical disease activity. This subclinical disease activity is not detectable using clinical and standard laboratory tests, and these patients will not reach stable remission off medication [37••]. Subclinical disease activity makes it difficult to determine when JIA patients have reached stable remission and treatment can be stopped. Therefore, a biomarker identifying patients at risk of disease relapse on the basis of the inflammatory status of their disease would be extremely valuable for improving paediatric medical care.

It has been suggested that continuation of MTX treatment for a longer period of time after clinical remission has been reached might reduce the risk of relapse after stopping treatment. Therefore, a randomised clinical trial was conducted to investigate whether longer treatment with MTX, for those who achieved clinical remission, could reduce relapse after MTX withdrawal, and which biomarkers could identify patients at higher risk of relapse after withdrawal. Patients were continuously treated with MTX for either six or 12 months after clinical remission was reached, and then followed after withdrawal of MTX. However, there was no significant difference in relapse between the two groups [37••]. On the basis of previously published results from this group, which revealed that MRP8/14 is a marker for subclinical disease activity [38, 39], they determined MRP8/14 levels in serum in both groups. Interestingly, patients with a high relapse rate after discontinuation of MTX had higher levels of MRP8/14 before stopping MTX than patients who did not flare. Thus, serum MRP8/14 levels could be a potential biomarker for predicting which patients will achieve stable remission after withdrawal of MTX, on the basis of inflammatory disease status [37••].

Similar results regarding the potential of MRP8/14 as a biomarker to identify patients at risk of relapse were described specifically for sJIA patients. Patients with new onset of active disease or with relapse had higher levels of serum MRP8/14, compared with patients who had reached stable remission. Measurements of MRP8/14 levels at time of stopping treatment revealed that, within up to six months, MRP8/14 levels were higher in patients with disease relapse than patients with no disease relapse. Prediction of patients at risk of relapse was highly accurate, with a sensitivity of 92 % and specificity of 88 % using MRP8/14 levels >740 ng mL^−1^ as a cut-off [20].

Another study investigated whether S100A12 and high sensitivity (hs) CRP could identify patients who have reached clinical remission on treatment, and are at risk of disease relapse after withdrawal of medication. High S100A12 and MRP8/14 levels were observed in patients who suffered disease relapse within six months of discontinuation of treatment. hsCRP levels did not differ between patients with disease relapse and those in remission, but this could be caused by the use of inclusion criteria based on normal hsCRP levels, because patients with high hsCRP levels are more prone to disease relapse. The patient group which relapsed within three months of stopping treatment had higher median levels of S100A12 and MRP8/14 compared with the group which relapsed later. S100A12, MRP8/14, and hsCRP were separately tested for their performance as biomarkers, and S100A12 was the best single biomarker for predicting disease relapse. The predictive performance could be improved by combining S100A12 with hsCRP [40].

Treatment strategies for JIA have the objective of full clinical remission [1], but only a very small number of patients remain in clinical remission, off medication, for long periods of time [41]. A recent study compared the transcriptional profiles of patients with active disease or those in clinical remission on medication (achieved by treatment with MTX or MTX plus TNF blockade) with the profiles of healthy children. Differences in transcriptional profile were observed between patients with active disease and those in clinical remission on medication, and also between those in remission off treatment and healthy children. These data suggest that being in remission does not mean a return to a “normal” inflammatory status, but rather a “disease-controlled” state [42]. Interestingly, the same group also demonstrated differences in transcriptional profile between patients in clinical remission induced by treatment with MTX or with MTX plus TNF inhibitor, and healthy individuals. This study confirmed previous findings from the group that the inflammatory status of patients in remission does not return to “normal”. Network analysis suggested a function for hepatocyte nuclear factor 4 alpha (HNF4α), which is expressed by T cells and granulocytes, in controlling genes associated with remission [43].

## Conclusions

Use of biomarkers for childhood arthritis is a rapidly expanding field. Such tests need to be reliable, simple to perform, economically feasible, and robust. In addition, they need to be tested and validated for large cohorts of children with JIA. Where proved reliable, biomarkers may assist with predicting disease type, course or severity, with predicting response to medication and therefore aiding treatment choices, and with accurate identification of children who can safely stop medication once apparent clinical remission is reached. The accurate choice of medicines most likely to work for each child, to enable the achievement of rapid remission for all, while avoiding unnecessary exposure to toxic drugs for those who do not need them and reducing unopposed inflammation to a minimum, is now the ultimate objective of modern management of arthritis. To move rapidly towards such ambitious objectives, multi-centre and international collaborations are needed to give every child with JIA the opportunity to take part in biomarker and cohort studies.
